# Supplementary material for: Gene flow between subpopulations of gray snapper (Lutjanus griseus) from the Caribbean and Gulf of Mexico
Source: PeerJ. 2020 Feb 10;8:e8485. doi: 10.7717/peerj.8485 (PMC7017790; doi:10.7717/peerj.8485)
Supplement: Supplemental Information 1 — Abbreviations: Campeche (C), Puerto de Veracruz (PV), Tuxpan (TX), Bahia de Chetumal (BC), Chiquilá (CH), Punta Herrero (PH) and Xahuaxol (X). [file peerj-08-8485-s001.docx]

| Site to | Site From | MLE | Lower | Upper |
| --- | --- | --- | --- | --- |
| *C* | *PV* | 2.2 | 1.7 | 2.9 |
| *C* | *TX* | 2.8 | 2.2 | 3.6 |
| *C* | *BC* | 3.7 | 3 | 4.5 |
| *C* | *CH* | 11.2 | 9.9 | 12.5 |
| *C* | *PH* | 2 | 1.5 | 2.7 |
| *C* | *X* | 3.4 | 2.7 | 4.1 |
| *PV* | *C* | 10 | 8.5 | 11.6 |
| *PV* | *TX* | 6.4 | 5.3 | 7.7 |
| *PV* | *BC* | 4 | 3 | 5 |
| *PV* | *CH* | 8.4 | 7 | 9.9 |
| *PV* | *PH* | 9.7 | 8.3 | 11.3 |
| *PV* | *X* | 3.5 | 2.7 | 4.5 |
| *TX* | *C* | 2.2 | 1.6 | 3 |
| *TX* | *PV* | 11.2 | 9.9 | 12.7 |
| *TX* | *BC* | 2.1 | 1.5 | 2.8 |
| *TX* | *CH* | 1.9 | 1.4 | 2.5 |
| *TX* | *PH* | 1.3 | 0.9 | 1.8 |
| *TX* | *X* | 2.1 | 1.5 | 2.7 |
| *BC* | *C* | 1.9 | 1.4 | 2.5 |
| *BC* | *PV* | 2.9 | 2.2 | 3.6 |
| *BC* | *TX* | 1.9 | 1.4 | 2.5 |
| *BC* | *CH* | 9.7 | 8.5 | 11.8 |
| *BC* | *PH* | 4 | 3.4 | 5.1 |
| *BC* | *X* | 4 | 3.1 | 4.8 |
| *CH* | *C* | 8.5 | 7.4 | 9.7 |
| *CH* | *PV* | 3.1 | 2.5 | 3.8 |
| *CH* | *TX* | 3 | 2.4 | 4 |
| *CH* | *BC* | 7.3 | 6.2 | 8.4 |
| *CH* | *PH* | 6.3 | 5.3 | 7.3 |
| *CH* | *X* | 5.1 | 5.3 | 6.1 |
| *PH* | *C* | 3.2 | 2.5 | 3.9 |
| *PH* | *PV* | 3.4 | 2.7 | 4.1 |
| *PH* | *TX* | 1.7 | 1 | 2.3 |
| *PH* | *BC* | 6.8 | 5.9 | 7.8 |
| *PH* | *CH* | 5.2 | 4.5 | 6.2 |
| *PH* | *X* | 8.4 | 7.2 | 9.6 |
| *X* | *C* | 1.7 | 1.1 | 2.2 |
| *X* | *PV* | 3 | 2.5 | 3.7 |
| *X* | *TX* | 2.8 | 2.3 | 3.5 |
| *X* | *BC* | 2 | 1.5 | 2.5 |
| *X* | *CH* | 3 | 2.4 | 3.6 |
| *X* | *PH* | 8.4 | 7.4 | 9.5 |
